# Tobacco retailer density and smoking behavior in a rural Australian jurisdiction without a tobacco retailer licensing system

**DOI:** 10.18332/tid/134190

**Published:** 2021-05-19

**Authors:** John Baker, Mohd Masood, Muhammad Aziz Rahman, Lukar Thornton, Stephen Begg

**Affiliations:** 1 Department of Community and Allied Health, La Trobe Rural Health School, La Trobe University, Bendigo, Australia; 2 Department of Dentistry and Oral Health, La Trobe Rural Health School, La Trobe University, Bendigo, Australia; 3 Institute of Dentistry, University of Turku, Turku, Finland; 4 Australian Institute for Primary Care and Ageing, La Trobe University, Melbourne, Australia; 5 School of Health, Federation University, Berwick, Australia; 6 Institute for Physical Activity and Nutrition, School of Exercise and Nutrition Sciences, Deakin University, Burwood, Australia

**Keywords:** smoking, policy, density, rural, licensing

## Abstract

**INTRODUCTION:**

An emerging body of research has developed around tobacco retailer density and its contribution to smoking behavior. This cross-sectional study aimed to determine the association between tobacco retailer density and smoking behavior in a rural Australian jurisdiction without a tobacco retailer licensing system in place.

**METHODS:**

A local government database (updated 2018) of listed tobacco retailers (n=93) was accessed and potential unlisted tobacco retailers (n=230) were added using online searches. All retailers (n=323) were visited in 2019 and GPS coordinates of retailers that sold tobacco (n=125) were assigned to suburbs in ArcMap. A community survey conducted in the Local Government Area provided smoking and sociodemographic data amongst adult respondents (n=8981). Associations between tobacco retailer density (calculated as the number of retailers per km^2^ based on respondents’ suburb of residence) and daily, occasional and experimental smoking were assessed using multilevel logistic regression analysis. Separate models with and without covariates were undertaken.

**RESULTS:**

Without adjusting for possible confounders, living in suburbs with greater retailer density did not increase the odds of daily smoking (OR=1.01; 95% CI: 0.92–1.12), occasional smoking (OR=1.05; 95% CI: 0.94–1.18), or experimental smoking (OR=0.98; 95% 0.92– 1.05). However, after adjustment, living in suburbs with greater retailer density increased the odds of occasional smoking behavior (AOR=1.37; 95% CI: 1.10–1.71) but not daily or experimental smoking.

**CONCLUSIONS:**

This study found a significant positive association between tobacco retailer density and the likelihood of occasional smoking in a rural Australian jurisdiction without a tobacco retailer licensing system in place. The findings strengthen calls for the introduction of a comprehensive, positive tobacco retailer licensing system to provide a framework for improving compliance with legislation and to reduce the overall availability of tobacco products in the community.

## INTRODUCTION

Tobacco smoking directly causes seven million deaths globally each year, while a further 1.2 million people die from exposure to secondhand smoke^1^. Current consumption patterns estimate that 400 million adults will die from smoking-related illnesses globally by 2050, and smoking will kill approximately 1 billion people by the end of the 21st century^2^. Despite this, tobacco products continue to be sold alongside everyday consumer products via an estimated 14 million points-of-sale (PoS) globally^3^. In response, an emerging body of research has developed around the retail availability of tobacco products and its contribution to a range of smoking behaviors including experimentation, uptake and continuation, and the undermining of cessation attempts amongst existing smokers who want to quit.

This literature has focused primarily on tobacco retailer density and how this might influence smoking behavior. Researchers have taken different approaches to the measurement of density, with some measuring the frequency of tobacco retailers located within circular buffers^4-6^ or polygons (using the street network or footpaths)^7-9^ at specified distances from geocoded locations (e.g. from participants’ homes). Other studies utilize Kernel Density Estimates (KDE) to generate continuous surface maps to model tobacco retailer density^10-12^, while others still have calculated the number of tobacco retailers within a defined area such as a census tract^13^. Several systematic reviews^14-17^ and a meta-analysis^18^ have documented statistically significant associations between tobacco retailer density and smoking behavior amongst both youth and adults, particularly around participants’ homes and activity spaces.

In Australia, researchers have explored associations between tobacco retailer density and sociodemographic characteristics at the neighborhood level, with several studies finding inverse correlations between density and socioeconomic status (SES)^12,19^, particularly in rural areas^20,21^. Other Australian research has explored tobacco retailer density and smoking behavior, with increased density associated with smoking behavior^4,12^. These studies tend to be undertaken in states or territories that have a tobacco retailer licensing or registration system in place, such as New South Wales, Tasmania and Western Australia^19,22^. Access to existing tobacco retailer databases, generated through licensing or registration systems, greatly facilitates such research and leads to greater accuracy when enumerating tobacco retailers^23,24^. Victoria is one of only two jurisdictions in Australia that currently does not have any type of licensing or registration for tobacco retailers^22^, making it difficult for researchers and policymakers to precisely map where the estimated 8000 tobacco retailers are located within this state^25^. It also raises questions around whether smoking behavior is influenced by the mostly unregulated retail availability of tobacco products in this jurisdiction.

To date, only one study has attempted to assess associations between tobacco retailer density and smoking behavior in Victoria, and this only examined certain business types within a 500 m radius of schools in a metropolitan student population^4^. Nevertheless, the results indicated that tobacco retailer density was associated with a significant increase in the number of cigarettes smoked during the previous seven days amongst students who indicated past-month smoking behavior, but not past-month smoking in the larger sample.

Thus, while previous research provides qualified evidence of an association between tobacco retailer density and smoking, at least in some settings in Australia, and a socioeconomic and geographical gradient in tobacco availability^20^, no studies to date have examined tobacco retailer density and its association with smoking behavior in a rural and regional Victorian setting, where the rates of smoking and socioeconomic disadvantage are generally higher than metropolitan areas^26,27^. The purpose of this study was to determine the association between tobacco retailer density and smoking behavior in a rural and regional population of Victoria.

## METHODS

This cross-sectional study was undertaken in a regional Local Government Area (LGA) in the State of Victoria, which, to preserve its identity, will be referred to as ‘Local Government X’. In Victoria there are 48 regional and rural LGAs, representing approximately 1.6 million people^28,29^. Local Government X was one of six LGAs that participated in the Healthy Heart of Victoria Active Living Census (ALC)^30^. Data collection for the ALC was conducted by an independent third party via an online survey and hardcopy questionnaire booklet between May and July 2019. A census-style approach was taken with respect to sampling, with all households in the region (n=224947 residents) being invited to participate and an overall response rate of 10.9% (n=24541). Microdata from the ALC were provided by Local Government X under a data sharing agreement.

### Smoking behavior

Respondents to the ALC, aged ≥18 years, were asked only one question in relation to smoking: ‘Which of the following best describes your smoking status?’, with possible responses including ‘smoke daily’, ‘smoke occasionally’, ‘don't smoke now but used to’, ‘tried a few times but never smoked regularly’ or ‘never smoked’. Responses were re-categorized in this analysis into dichotomous outcome variables (yes/no) for each of the following outcomes of interest: ‘Daily smoker’, ‘Occasional smoker’, and ‘Experimental smoker’ (tried a few times but never smoked regularly). Respondents aged <18 years were not asked about their smoking behavior.

### Individual-level covariates

Other ALC variables included in the analysis as individual-level covariates were age (18–34, 35–49, 50–69 and ≥70 years), sex (male or female), self-reported health status (poor, fair, good, very good or excellent), self-reported financial position (very poor/poor, just getting along, reasonably comfortable or very comfortable/prosperous), self-reported education level (Bachelor’s or higher, completed Year 12, or did not complete Year 12), self-reported alcohol consumption (daily, less than daily or does not drink), and whether or not respondents identified as an Aboriginal or Torres Strait Islander (ATSI).

Only ALC respondents who provided valid responses to each of the above questions and who also indicated that their suburb of residence was a valid suburb of Local Government X were included in the analysis. A total of 1845 respondents (17.0%) aged ≥18 years were excluded due to missing values.

### Tobacco retailer density

Suburb of residence was the most specific geographical identifier in the ALC; tobacco retailer density was therefore determined at the suburb level by dividing the number of confirmed tobacco retailers in a respondent’s suburb of residence (as enumerated below) by the geographical area of that suburb in km^2^. Suburbs in this context are officially gazetted boundaries of suburbs in cities and larger towns, and localities elsewhere^31^. In Victoria, there were approximately 2672 suburbs in 2016^32^. The geographical area of each suburb was determined in ArcMap.

An existing database maintained by Local Government X of known tobacco retailers within the municipality (updated in April 2018) was obtained via a Freedom of Information request after sensitive or personal information and enforcement-related information had been redacted. Duplicate listings were removed and internet searches were undertaken between May and August 2019 to identify additional businesses that might also sell tobacco within the municipality. All businesses from the original database (n=93) and from internet sources (n=230) were visited between June and August 2019 by the primary researcher who posed as a potential customer. Visual cues (e.g. observing signage such as a price board, a cigarette gantry or working vending machine) or verbal confirmation (e.g. asking the sales assistant) were used to determine whether a business currently sold tobacco. For businesses that only opened seasonally, were geographically distant, or only operated at night, verbal confirmation was attempted via telephone. Four of these retailers were subsequently telephoned by the researcher to determine whether tobacco was sold, and two potential retailers were identified during field visits to other retailers.

A positive assessment was made at a physical premise in which tobacco could be purchased by the general public either at a staffed PoS or through a working vending machine. Excluded from this definition were telephone or internet-based businesses, home-delivery businesses and wholesalers. The exact coordinates of each business confirmed as a tobacco retailer were recorded on site and its suburb was determined in ArcMap. Further details on these methods are available elsewhere^33^.

### Suburb-level socioeconomic status

Suburb-level socioeconomic status was derived from the 2016 Australian Bureau of Statistics Index of Relative Socio-Economic Disadvantage (IRSD)^32^. Raw IRSD scores were recategorized into quintiles such that there were equal numbers of suburbs in each quintile. Respondents were assigned an IRSD quintile on the basis of their suburb of residence.

### Statistical analysis

Means and standard deviations for tobacco retailer density and frequencies and percentages for each of the covariates were used for descriptive analyses. Given the census-style sampling approach of the ALC, Intraclass Correlations (ICC) were used to assess the extent of clustering of each outcome of interest (daily smoker, occasional smoker and experimental smoker) within households (using the household ID of respondents) and suburbs (using their suburb of residence). The likelihood of each outcome dependent on tobacco retailer density was assessed using single- or multi-level logistic regression analysis, as appropriate. Separate models for each outcome were conducted without and with covariates (Models 1 and 2, respectively). All analyses were conducted using Stata (V15.1).

## RESULTS

A total of 8981 respondents were included in the analysis, including 536 daily smokers, 234 occasional smokers and 949 experimental smokers (Table 1). Means and standard deviations for tobacco retailer density are reported across the different smoking behaviors, while numbers and percentages are reported for the remaining categorical variables.

**Table 1:** Tobacco retailer density, sociodemographic and behavioural attributes of respondents

	*Daily smoker (n=536)*	*Occasional smoker (n=234)*	*Experimental smoker (n=949)*	*Ex and never smoker (n=7262)*	*Total (n=8981)*
	*n (%)*	*n (%)*	*n (%)*	*n (%)*	*n (%)*
**Tobacco retailers per km^2^ ,** mean (SD)	1.00 (1.28)	1.08 (1.40)	0.98 (1.33)	0.99 (1.39)	0.99 (1.38)
**Suburb-level IRSD quintile**					
Most disadvantaged	217 (40.4)	74 (31.6)	215 (22.6)	1912 (26.3)	2418 (26.9)
Q2	113 (21.0)	44 (18.8)	221 (23.2)	1350 (18.5)	1728 (19.2)
Q3	85 (15.8)	37 (15.8)	172 (18.1)	1341 (18.4)	1635 (18.2)
Q4	70 (13.0)	45 (19.2)	180 (18.9)	1438 (19.8)	1733 (19.3)
Least disadvantaged	51 (9.5)	34 (14.5)	161 (16.9)	1221 (16.8)	1467 (16.3)
**Health status**					
Excellent	16 (2.9)	24 (10.2)	127 (13.3)	867 (11.9)	1034 (11.5)
Very good	117 (21.8)	75 (32.0)	378 (39.8)	2648 (36.6)	3218 (35.8)
Good	240 (44.7)	95 (40.6)	316 (33.3)	2544 (35.0)	3195 (35.5)
Fair	117 (21.8)	34 (14.5)	106 (11.1)	952 (13.1)	1209 (13.4)
Poor	46 (8.5)	6 (2.5)	22 (2.3)	251 (3.4)	325 (3.6)
**Age** (years)					
18–34	127 (23.6)	88 (37.6)	353 (37.2)	1418 (19.5)	1986 (22.1)
35–49	159 (29.6)	70 (29.9)	247 (26.0)	1658 (22.8)	2134 (23.7)
50–69	221 (41.2)	63 (26.9)	275 (28.9)	2826 (38.9)	3385 (37.6)
≥70	29 (5.4)	13 (5.5)	74 (7.8)	1360 (18.7)	1476 (16.4)
**Sex**					
Male	243 (45.3)	119 (50.8)	399 (42.4)	3082 (42.4)	3843 (42.7)
Female	293 (54.6)	115 (49.1)	550 (57.9)	4180 (57.5)	5138 (57.2)
**Financial position**					
Prosperous/very comfortable	42 (7.8)	32 (13.6)	178 (18.7)	1325 (18.2)	1577 (17.5)
Reasonably comfortable	235 (43.8)	118 (50.4)	544 (57.3)	4151 (57.1)	5048 (56.2)
Just getting along	215 (40.1)	73 (31.2)	204 (21.5)	1608 (22.1)	2100 (23.3)
Poor/very poor	44 (8.2)	11 (4.7)	23 (2.4)	178 (2.4)	256 (2.8)
**Education level**					
Bachelor’s or higher	135 (25.1)	75 (32.0)	497 (52.3)	3252 (44.7)	3959 (44.0)
Completed Year 12	215 (40.1)	107 (45.7)	313 (32.9)	2489 (34.2)	3124 (34.7)
Did not complete Year 12	186 (34.7)	52 (22.2)	139 (14.6)	1521 (20.9)	1898 (21.1)
**Alcohol consumption**					
Daily	84 (15.6)	22 (9.4)	55 (5.8)	528 (7.2)	689 (7.6)
Less than daily	361 (67.3)	194 (82.9)	805 (84.8)	5292 (72.8)	6652 (74.0)
Does not drink	91 (16.9)	18 (7.6)	89 (9.3)	1442 (19.8)	1640 (18.2)
**ATSI status**					
Aboriginal or Torres Strait Islander	17 (3.1)	4 (1.7)	13 (1.3)	56 (0.7)	90 (1.0)
Does not identify as ATSI	519 (96.8)	230 (98.2)	936 (98.6)	7206 (99.2)	8891 (99.0)

ISRD: index of relative socioeconomic disadvantage. ATSI: Aboriginal or Torres Strait Islander. Daily smoker: smokes daily. Occasional smoker: smokes occasionally. Experimental smoker: tried a few times but never smoked regularly. Ex and never smoker: does not smoke now but used to and never smoked, combined.

Clustering was observed for each outcome of interest (daily smoking, occasional smoking and experimental smoking) within households (ICC of 0.60, 0.54 and 0.40, respectively) but not suburbs (ICC of 0.07, 0.02 and 0.01, respectively). Multilevel logistic regression clustering on households was therefore used in subsequent analyses. None of the associations between tobacco retailer density and two of the outcomes of interest (daily smoking and experimental smoking) were statistically significant in either the bivariate or multivariate models (Model 1 and Model 2 in Table 2, respectively). However, the insignificant association between tobacco retailer density and occasional smoking in the bivariate model (Model 1, Table 2: OR=1.05; 95% CI: 0.94– 1.18) became statistically significant after adjusting for covariates (Model 2, Table 2: AOR=1.37; 95% CI: 1.10–1.71), suggesting a degree of confounding between tobacco retailer density and occasional smoking that was not apparent with the other outcomes.

**Table 2:** Tobacco retailer density, sociodemographic and behavioural attributes of respondents and the likelihood of different smoking behaviours

	*Outcome 1 Daily smoker ^a^*		*Outcome 2 Occasional smoker ^b^*		*Outcome 3 Experimental smoker ^c^*	
	*OR*	*95% CI*	*OR*	*95% CI*	*OR*	*95% CI*
**Model 1: Unadjusted ORs**						
Tobacco retailers per km^2^	1.01	(0.92–1.12)	1.05	(0.94–1.18)	0. 98	(0.92–1.05)
**Model 2: Adjusted ORs**						
Tobacco retailers per km^2^	1.08	(0.93–1.26)	1.37*	(1.10–1.71)	0.98	(0.89–1.08)
**IRSD**						
Most disadvantaged	Ref.		Ref.		Ref.	
2	0.73	(0.50–1.07)	0.68	(0.41–1.15)	1.33*	(1.02–1.73)
3	0.53*	(0.30–0.94)	0.29*	(0.12–0.71)	1.10	(0.75–1.60)
4	0.44*	(0.29–0.68)	0.97	(0.58–1.63)	1.11	(0.84–1.46)
Least disadvantaged	0.35*	(0.21–0.56)	0.86	(0.48–1.53)	1.14	(0.85–1.52)
**Health status**						
Excellent	Ref.		Ref.		Ref.	
Very good	2.54	(1.32–4.91)	0.99	(0.55–1.78)	0.99	(0.75–1.30)
Good	5.97*	(3.08–11.56)	1.26	(0.70–2.26)	0.86	(0.65–1.15)
Fair	7.80*	(3.84–15.83)	1.18	(0.59–2.38)	0.87	(0.61–1.23)
Poor	11.55*	(5.09–26.20)	0.69	(0.22–2.15)	0.71	(0.40–1.28)
**Age** (years)						
18–34	Ref.		Ref.		Ref.	
35–49	1.41	(0.98–2.03)	0.64	(0.41–0.99)	0.55*	(0.44–0.69)
50–69	0.92	(0.66–1.29)	0.31*	(0.19–0.48)	0.36*	(0.29–0.46)
≥70	0.14*	(0.08–0.26)	0.12*	(0.05–0.26)	0.23*	(0.16–0.32)
**Sex**						
Male	Ref.		Ref.		Ref.	
Female	0.93	(0.73–1.18)	0.65*	(0.47–0.90)	1.00	(0.85–1.18)
**Financial position**						
Prosperous/very comfortable	Ref.		Ref.		Ref.	
Reasonably comfortable	1.41	(0.88–2.27)	1.03	(0.61–1.74)	0.96	(0.76–1.23)
Just getting along	3.04*	(1.82–5.08)	1.59	(0.88–2.87)	0.93	(0.69–1.24)
Poor/very poor	4.58*	(2.19–9.56)	2.68	(0.98–7.31)	0.99	(0.54–1.81)
**Education level**						
Bachelor’s or higher	Ref.		Ref.		Ref.	
Completed Year 12	2.08*	(1.51–2.84)	1.97*	(1.33–2.91)	0.77*	(0.64–0.94)
Did not complete Year 12	2.86*	(2.01–4.06)	1.82*	(1.12–2.95)	0.68*	(0.53–0.87)
**Alcohol consumption**						
Daily	Ref.		Ref.		Ref.	
Less than daily	0.25*	(0.16–0.37)	0.75	(0.41–1.37)	1.23	(0.86–1.75)
Does not drink	0. 18*	(0.11–0.30)	0.25*	(0.11–0.55)	0.53*	(0.35–0.82)
**ATSI status**						
Aboriginal or Torres Strait Islander	Ref.		Ref.		Ref.	
Does not identify as ATSI	0.29*	(0.11–0.73)	1.22	(0.28–5.21)	0.64	(0.30–1.38)

IRSD: index of relative socioeconomic disadvantage, ATSI: Aboriginal or Torres Strait Islander. Daily smoker: smokes daily. Occasional smoker: smokes occasionally. Experimental smoker: tried a few times but never smoked regularly.

a Daily smokers vs all other respondents.

b Occasional smokers vs all other respondents.

c Experimental smokers vs all other respondents.

* p<0.05.

The associations between the outcomes of interest and the covariates were mostly in expected directions (Model 2, Table 2). For example, the odds of reporting daily smoking behavior significantly increased with decreasing self-reported health status and financial position, while the odds of reporting occasional or experimental smoking behavior decreased with age, with those aged ≥70 years much less likely than those aged 18–35 years to report any of the smoking behaviors. Similarly, the odds of reporting daily or occasional smoking behavior increased as the level of education decreased, although the reverse association was observed for experimental smoking. Females were less likely than males to report occasional smoking, abstainers were less likely than drinkers to report any of the smoking behaviors, and those who did not identify as Aboriginal or Torres Strait Islander were less likely to report daily smoking than those who did.

## DISCUSSION

This study was conducted in a regional area of Victoria, which is characterized by higher than average rates of smoking, particularly amongst adolescents, and greater levels of socioeconomic disadvantage^26,34^. It found that tobacco retailer density was associated with occasional smoking behavior, but not daily or experimental smoking behaviors. This finding is consistent with studies focusing on adults in other settings, which have found significant positive associations between tobacco retailer density and current or occasional smoking behavior^35,36^.

The findings are also consistent with research involving a similarly disadvantaged population of regional New South Wales^37^, which found that nearly three-quarters of ‘current smokers’ (defined as daily, weekly or occasional smokers) reported having a tobacco retailer within walking distance of home, and that younger ‘current smokers’ were significantly more likely to report the presence of a tobacco retailer nearby. Research in a rural population of the United States^38^ also identified an association between ‘current smokers’ (defined as daily or occasional smoking) and the retail availability of tobacco.

There is evidence in the literature of a strong association between tobacco retailer density and suburb-level socioeconomic status. Research in Western Australia, for example, has found that suburbs and towns with low socioeconomic status have more than four times the number of tobacco retailers, compared to high socioeconomic status suburbs in both metropolitan and regional areas^20^. Similar research in Tasmania found a disproportionate concentration of tobacco retailers in regional and remote areas and in low socioeconomic areas^19^. This is consistent with the finding that the tobacco industry actively targets poorer communities to market and sell its products^39,40^.

A possible explanation for reported associations between tobacco retailer density and smoking behavior, therefore, is that smokers tend to live in lower socioeconomic suburbs and lower socioeconomic suburbs tend to have higher tobacco availability.

### Strengths and limitations

A key strength of the present study is that both individual-level and suburb-level socioeconomic status were controlled for in the analysis thus allowing for the association between tobacco retailer density and smoking behavior to be observed independently from these factors. Interestingly, in this sample it was only after controlling for these and the other covariates that the association between tobacco retailer density and occasional smoking became significant, suggesting a degree of confounding in the bivariate analysis for this outcome.

A further strength of this study is that it relied on a robust method for enumerating tobacco retailers and hence tobacco retailer density in an environment without a tobacco retailer licensing system in place. This involved physically visiting or contacting (via telephone) a large number of potential retailers, which was both time and resource intensive. It is possible that false-positive or false-negative attributions may have occurred during the site visits; for example, retailers may have had visual cues to indicate the sale of tobacco (e.g. signage or a cigarette gantry) but no longer sold tobacco, while other retailers may not have had any visual cues to indicate the sale of tobacco but did actually sell. However, this misattribution was unlikely to have been systematic or result in bias.

The main limitation of the present study was that it relied on secondary data to measure smoking behavior. As respondents’ precise residential location was not collected in the ALC, an individual’s exposure to tobacco retailers could only be determined by which suburb they lived in. Previous studies have accessed individual participants’ geocoded location (e.g. participants’ home, school or both), which allows for a more precise measure of the availability of tobacco and its association with smoking behaviors. It is possible that the lack of geographical specificity in the present study diluted the strength of the association between tobacco retailer density and smoking reported here. The ALC also restricted the single smoking behavior question to respondents aged ≥18 years. Given previous research indicates most people who experiment with smoking do so before the age of 18 years^41^, the prevalence of experimental smoking is likely to be under-represented in the ALC. One recommendation arising from the present study therefore is that more precise geographical identifiers and more detailed questions about smoking behaviors at all ages be considered in future iterations of the ALC.

Other limitations of the present study include the cross-sectional nature of the study design, which means that causation between exposures and outcomes cannot be inferred from this analysis, and the relatively small numbers of respondents who reported daily, occasional or experimental smoking overall, meaning that the results should be interpreted with caution.

A number of jurisdictions both within Australia and internationally have implemented legislation to allow for the licensing of tobacco retailers. A ‘positive’ tobacco retailer licensing system requires retailers to register with a government authority by paying an annual fee. It is the foundation of a robust framework for ensuring compliance with existing tobacco sales legislation through the creation of accurate databases of active retailers in a given area and the generation of revenue for regular compliance checks, education visits and underage test purchasing activities^42^. A key objective of improved compliance is to reduce the likelihood of sales to minors and prevent experimentation and initiation in this group^42,43^. A recent study in regional Victoria found that, in the absence of a licensing system, a large proportion of tobacco retailers are likely to be operating without formal oversight from local authorities whose responsibility is to ensure compliance^33^.

The other main advantage of a positive tobacco retailer licensing system is that it provides a mechanism for regulating tobacco availability in a community by influencing how many retailers there are and where they are allowed to operate. A study in South Australia, for example, found a 23.7% decrease in licence uptake and renewal simply by increasing the licensing fee from $A12 to $A200 per year^44^. San Francisco has gone a step further by implementing legislation to cap the number of tobacco retailer licences available per suburb, and to prohibit retailers from selling tobacco within 150 m of a school or another retailer^45,46^. This legislation will gradually reduce the total number of retailers from approximately 1001 to 495 retailers in that city. A key recommendation from the present study therefore is that jurisdictions, including the state of Victoria, adopt international best practice by introducing comprehensive positive tobacco retailer licensing systems to improve retailer compliance with existing tobacco sales legislation and to work towards reducing tobacco availability in the community.

## CONCLUSIONS

The current study found a significant positive association between tobacco retailer density and the likelihood of occasional smoking in a regional population of Victoria without a tobacco retailer licensing system in place. The findings strengthen calls for the introduction of a comprehensive, positive tobacco retailer licensing system to provide a framework for improving tobacco retailer compliance with legislation and reducing the overall retail availability of tobacco products in the community.
